# *Bifidobacterium longum* subsp. *longum* Reduces Perceived Psychological Stress in Healthy Adults: An Exploratory Clinical Trial

**DOI:** 10.3390/nu15143122

**Published:** 2023-07-13

**Authors:** Marcus Boehme, Noëla Rémond-Derbez, Clara Lerond, Luca Lavalle, Sonia Keddani, Myriam Steinmann, Andreas Rytz, Boushra Dalile, Kristin Verbeke, Lukas Van Oudenhove, Pascal Steiner, Bernard Berger, Maria Vicario, Gabriela Bergonzelli, Sara Colombo Mottaz, Julie Hudry

**Affiliations:** 1 Nestlé Institute of Health Sciences, Société des Produits Nestlé S.A., 1000 Lausanne 26, Switzerland; noela.remond-derbez@rd.nestle.com (N.R.-D.); sonia.keddani@rd.nestle.com (S.K.); myriam.steinmann@rdls.nestle.com (M.S.); pascal.steiner1@rd.nestle.com (P.S.); bernard.berger@rdls.nestle.com (B.B.); maria.vicarioperez@rd.nestle.com (M.V.); julie.hudry@rdls.nestle.com (J.H.); 2 Clinical Research Unit, Société des Produits Nestlé S.A., 1000 Lausanne 26, Switzerland; luca.lavalle1@rd.nestle.com (L.L.); andreas.rytz@rdls.nestle.com (A.R.); sara.colombomottaz@rd.nestle.com (S.C.M.); 3 Translational Research Center in Gastrointestinal Disorders (TARGID), Department of Chronic Diseases and Metabolism, Faculty of Medicine, KU Leuven, 3000 Leuven, Belgium; boushra.dalile@kuleuven.be (B.D.); kristin.verbeke@kuleuven.be (K.V.); lukas.vanoudenhove@kuleuven.be (L.V.O.); 4 Leuven Brain Institute, KU Leuven, 3000 Leuven, Belgium

**Keywords:** probiotic, psychological stress, sleep, cortisol, gut–brain axis

## Abstract

Emerging science shows that probiotic intake may impact stress and mental health. We investigated the effect of a 6-week intervention with *Bifidobacterium longum (BL) NCC3001* (1 × 10^10^ CFU/daily) on stress-related psychological and physiological parameters in 45 healthy adults with mild-to-moderate stress using a randomized, placebo-controlled, two-arm, parallel, double-blind design. The main results showed that supplementation with the probiotic significantly reduced the perceived stress and improved the subjective sleep quality score compared to placebo. Comparing the two groups, momentary subjective assessments concomitant to the Maastricht Acute Stress Test revealed a lower amount of pain experience in the probiotic group and a higher amount of relief at the end of the procedure in the placebo group, reflected by higher scores in the positive affect state. The awakening of the salivary cortisol response was not affected by the intervention, yet the reduction observed in the salivary cortisol stress response post-intervention was higher in the placebo group than the probiotic group. Multivariate analysis further indicated that a reduction in perceived stress correlated with a reduction in anxiety, in depression, and in the cortisol awakening response after the 6-week intervention. This exploratory trial provides promising insights into *BL NCC3001* to reduce perceived stress in a healthy population and supports the potential of nutritional solutions including probiotics to improve mental health.

## 1. Introduction

Psychosocial stress is a significant and increasing burden nowadays in our society, is a risk factor for mood disorders including depression and sleep disorders [1], and can further cause gut discomfort [2]. There is mounting evidence to suggest that nutritional interventions can influence our stress responses [3]. One of the routes by which nutrition can influence physiological and psychological stress responses involves the microbiota–gut–brain axis [2,4].

Dietary interventions, such as the intake of pre- and probiotics, can modulate the microbiota–gut–brain axis. Probiotics, live microorganisms that provide health benefits when consumed in adequate amounts [5], were proposed as a nutritional approach to impact the physiological functions related to emotion regulation, in particular for reducing psychological stress and stress-related symptoms [6]. These findings sparked interest in the potential of probiotics, notably the *Bifidobacterium* and *Lactobacillus* strains, to help individuals in managing stress and stress-related symptoms. Previously, we showed that a 6-week intervention with the *Bifidobacterium longum (BL) strain NCC3001* significantly improved depression scores, in association with a decreased emotional reaction to fearful stimuli in non-constipated patients with irritable bowel syndrome (IBS) [7]. Furthermore, in a randomized, crossover study with dogs displaying anxious behavior, we showed a significant reduction in the stress response following mild acute stressors, as measured by reduced salivary cortisol and increased heart rate variability (HRV), an indicator of reduced psychosocial stress, following a 6-week intervention with the same *BL NCC3001* [8]. Preclinical assessments showed that the effect of *BL NCC3001* on anxiety is mediated through the microbiota–gut–brain axis and vagal signaling. Indeed, a study with vagotomized mice showed that the anxiolytic effect of *BL NCC3001* requires vagus nerve integrity [9]. Moreover, *BL NCC3001* restored, back to control values, the infection-induced reduction in expression of the hippocampal mRNA levels of brain-derived neurotrophic factor (BDNF), a neurotrophin linked to depression and stress [10].

Based on the effects we observed with *BL NCC3001*, especially in the population with IBS, which is characterized by a high depression prevalence [11], we hypothesized that a healthy population at risk for stress-related mental health disturbances benefits from *BL NCC3001*, which positively modulates stress response and mood parameters. Therefore, we designed an exploratory clinical trial to investigate the effect of *BL NCC3001* on stress-related psychological and physiological parameters and in response to acute stress in healthy adults who typically experience mild-to-moderate levels of stress.

## 2. Materials and Methods

### 2.1. Study Design and Population

**Figure 1:**
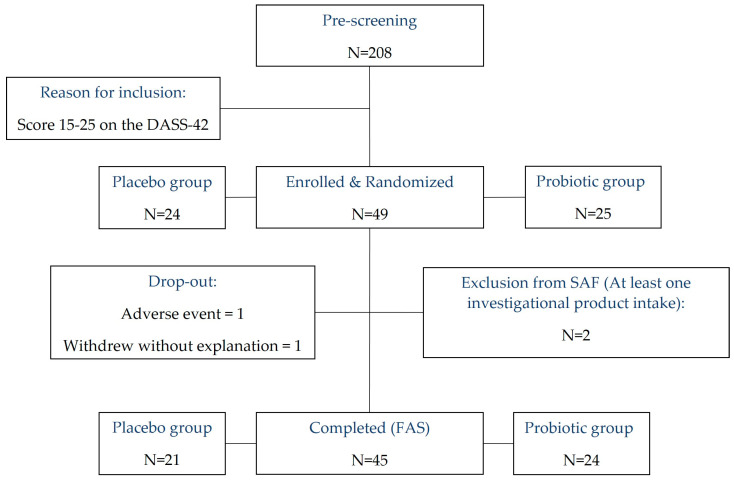
CONSORT flowchart depicting the progression of participants through the study. DASS-42, Depression, Anxiety and Stress Scale; SAF, Safety Analysis Set; FAS, Full Analysis Set.

This randomized, placebo-controlled, 2-arm, parallel, double-blind exploratory clinical trial was conducted in Switzerland between December 2020 and August 2021. Being an exploratory trial, no a priori sample size calculation was performed. Out of a total of 49 eligible volunteers, 47 completed the study (see Figure 1). Inclusion criteria were mild-to-moderate psychological stress level (stress score 15–25 on the Depression, Anxiety and Stress Scale; DASS-42), age in the range of 25–65 years, and BMI in the range of 18.5–29.9 kg/m^2^. Exclusion criteria were diagnosis of food allergies, organic gastrointestinal pathology, chronic diarrhea, concurrent systemic chronic disease (e.g., endocrine, cardiovascular, metabolic, liver, or celiac disease), adherence to an overly unbalanced diet (e.g., vegan, paleo, ketogenic, or raw diets), and other conditions that may affect mood (e.g., psychoactive drugs, pregnancy, heavy coffee or alcohol consumption, or lactating women). All subjects provided written informed consent for their participation. The study protocol (registered under NCT05226520) was approved by the Ethics Committee of Canton de Vaud. Participants were randomly allocated to one of the following groups: probiotic (*n* = 25) or placebo (*n* = 24). Randomization was carried out using Medidata RTSM. Block randomization was employed and included the stress levels (mild and moderate) measured from the DASS-42 questionnaire and gender as stratification factors. Participants’ characteristics are given in Table 1.

### 2.2. Intervention

Products were supplied in the form of dry powder contained in aluminum sachets. In alignment with previous preclinical and clinical trials [7,9,10], the probiotic product contained 1 × 10^10^ colony-forming units (CFUs) of *BL NCC3001* in maltodextrin per sachet, and the placebo contained yeast extract, cysteine HCl, and pea flour in maltodextrin to match the flavor and taste profile of the probiotic product. Participants consumed one sachet per day for six weeks at breakfast by mixing the powder in about 50 mL of water.

### 2.3. Procedure

The experimental procedure comprised two separate testing visits (visit 2: baseline; visit 3: post-intervention) performed at baseline and after 6 weeks starting at 1:00 p.m. following a 24 h abstention from alcohol, coffee (caffeinated or decaffeinated), other caffeinated products, or any other stimulant. Prior to the test visits, participants collected a fecal sample at home and awakening saliva samples as previously described [12]. During the test visits, upon arrival in the laboratory, participants completed the Perceived Stress Scale (PSS), the Hospital Anxiety and Depression Scales (HAD-A and HADS-D), the Gastrointestinal Symptom Rating Scale (GSRS), and the Pittsburgh Sleep Quality Index (PSQI) questionnaire. Psychological stress was assessed with the Perceived Stress Scale (PSS-14; scores 0–56) [13], sleep quality with the Pittsburg Sleep Quality Index (PSQI; global scores 0–21) [14], anxiety with the Hospital Anxiety Scale (HAD-A; scores 0–21), depression with the Hospital Depression Scale (HAD-D; scores 0–21) [15] and gut comfort with the Gastrointestinal Symptom Rating Scale (GSRS) [16] at baseline (visit 2) and after 6-week intervention (visit 3). The PSS-14 represents a classic self-reported questionnaire measuring individual stress levels during the previous month [13], which is validated for adequate psychometric properties [17]. Notably, PSS-14 is not a diagnostic tool with standard cut-off scores. However, individual total scores are usually categorized as low stress (scores 0–18), moderate stress (scores 19–37), and high stress (scores 38–56). The PSQI is a validated 19-item, self-rated questionnaire which assesses sleep quality and disturbance over the past month in clinical populations [14]. The global PSQI score is calculated as the sum of seven component scores (subjective sleep quality, sleep latency, sleep duration, habitual sleep efficiency, sleep disturbances, use of sleeping medication, and daytime dysfunction) weighted on a 0–3 interval scale, where lower scores denote better sleep quality. Overall, the global scores range from 0 to 21, and a score of 5 or above is considered as a significant sleep disturbance. The Hospital Anxiety and Depression Scale (HADS) is a validated widely used self-administered questionnaire for clinical practice and research [15]. It has the capacity to detect anxiety and depressive disorders and track their evolution. The GSRS comprises five subscales (Reflux, Diarrhea, Constipation, Abdominal Pain, and Indigestion Syndrome). Subscale scores range from 1 to 7, and higher scores represent more discomfort.

Subsequently, participants performed the Maastricht Acute Stress Test (MAST) according to procedure described in Smeets et al. (2012). In brief, the MAST consisted in a first 5 min period of instruction (stress anticipation), followed by a 10 min period of physical (hand immersion in cold water), with cognitive (mental arithmetic trials) and social (videotaping) stressors alternately administered by a trained experimenter [18]. Participants provided salivary samples and completed momentary ratings using the Positive and Negative Affect Schedule (PANAS) [19], the State Trait Anxiety Inventory 6-item (STAI-6) short form [20], and the Visual Analog Scales (VAS, which measures pain intensity) immediately before and after the MAST performed at baseline and after 6-week intervention (see Supplementary Table S1). Positive and negative affect scores (1–50) were computed as the summed scores of the 10 positive and 10 negative items of the PANAS, respectively. Three 100 mm VAS, anchored from “not at all” to “extremely”, were used to assess subjective perception of the stress induction task on stressful, painful, and uncomfortable dimensions, as previously described [12,18,21]. Heart rate (HR) and skin conductance were continuously monitored throughout the MAST procedure.

### 2.4. Salivary Cortisol

Salivary cortisol awakening response was quantified at baseline and after 6-week supplementation as an index of the hypothalamic–pituitary–adrenal (HPA) axis activity in relation to chronic stress [22]. Five saliva samples were collected at home by the participants according to the previously described procedure [23]: one sample immediately upon waking and subsequently every 15 min until 1 h post-waking. Samples were transported to the laboratory in a cool container and were stored at −80 °C. In addition, salivary cortisol was collected in conjunction with the MAST procedure at baseline and after the 6-week supplementation period. Saliva samples were collected before the stressor (t − 20), immediately after (t + 0), and at 5 subsequent time points after the physical stressor during a 40 min recovery period (t + 5; t + 10; t + 20; t + 30; t + 40). All saliva samples were collected using commercial Salivette tubes (Sarstedt Ltd., Nümbrecht, Germany) and then stored at −80 °C prior to assessment of free cortisol concentration. Saliva samples were thawed and centrifuged for 2 min at 1000× *g* and 10 min at 2500× *g* without the swab to remove particulate material. Subsequently, free cortisol was quantified after randomization of the samples, using the saliva cortisol ELISA kit (RE52611, IBL International GmbH, Hamburg, Germany), in accordance with the instructions of the manufacturer.

### 2.5. Autonomic Parameters

Autonomic parameters were collected by means of a portable-computer-based data acquisition system with AcqKnowledge software (BIOPAC Systems Inc., Camino Goleta, CA, USA), using a finger photoplethysmograph and electrodermal activity (EDA) transducers. They were placed on the nondominant hand of the participants. The photoplethysmograph provided data for heart rate and heart rate variability (HRV) that were analyzed with Kubios HRV software (Kubios software, Premium 3.3.1, Finland). HRV parameters of interest were the root mean square of successive differences between normal heartbeats in millisecond (RMSSD), the mean of heart rate (Mean HR), the peak in the low-frequency band (LF) (0.04–0.15 Hz), the peak in the high-frequency band (HF) (0.15–0.4 Hz), and the ratio of low-frequency to high-frequency peaks (LF:HF). The electrodermal sensor provided the Skin Conductance Level (SCL) in microsiemens. SCL was processed with AcqKnowledge software using a smoothing baseline removal with a window width of 2 s, a low-pass filter with a cutoff frequency of 1 Hz, and a linear interpolation method. The waveform was resampled to 7.813 Hz, and phasic EDA was subtracted from the signal. Both cardiac and electrodermal data were computed for periods of different lengths. They correspond to 5 min baseline before instructions were given (Before), 5 min stress anticipation (during the instructions; During 1), the average of cold hand immersions (During 2), the average of mental arithmetic trials (During 3), and 8 periods of rest after the stressor (After 1; After 2; After 3; After 4; After 5; After 6; After 7; After 8), as shown in Supplementary Table S1.

### 2.6. Fecal BL NCC3001 Abundance Analysis

Fecal *BL NCC3001* abundance analysis was performed as previously described (Martin et al., *submitted*). Briefly, fecal DNA was extracted with the kit QIAamp FAST DNA Stool Mini Kit (51604, Qiagen, Hilden, Germany). DNA concentrations were measured using the PicoGreen fluorescence method (Thermo Fisher, Waltham, MA, USA). A chromosomic region, spanning an insertion site of a mobile element, was previously used to specifically detect the strain *BL NCC3001* [9]. A TaqMan MGB assay targeting this strain-specific region was designed (Primer Express 3.0, Applied Biosystems, Waltham, MA, USA) to measure the abundance of the probiotic in fecal samples (BL NCC3001_Fw 5′-GTGATAACCTCAACAACCGACAAC-3′, BL NCC3001_Pr (FAM) 5′- ATCTGCCCTTAACGGC-3′ (MGB), and BL NCC3001_Rev 5′- GCATCACCTCGTTCTCGACAA-3′). The MasterMix LightCycler^®^ 1536 DNA Green Master (05573092001, Roche) was used with a final concentration of 0.9 µM of each primer and 0.25 µM of the probe. Each data point was run as technical triplicates, and a standard curve was built in serial 10-fold dilution of *BL NCC3001* genomic DNA. The assay was performed on an LC480 II cycler (Roche) using the following PCR conditions: 7 min at 95 °C for Taq activation, 10 s at 95 °C for denaturation, 30 s at 60 °C for annealing and extension × 40 cycles, and then cooling 30 s at 40 °C. A further TaqMan MGB assay was used to normalize the *BL NCC3001* abundance relative to the bacterial load [24].

### 2.7. Statistical Analysis

Statistical analyses were conducted on 45 subjects that were randomized to one of the two arms (*BL NCC3001* or placebo), which received at least one dose of product and provided post-randomization data.

Psychological endpoints related to acute stress (PANAS, STAI-6, and VAS) and general assessments (PSS-14, HADS, GSRS, and PSQI) were used to assess the effect of the intervention by estimating the treatment difference (probiotic vs. placebo) at the post-intervention visit by an analysis of covariance (ANCOVA) model including treatment as independent variable as well as the three covariates: gender, DASS-42, and the pre-treatment value considered as baseline.

Since saliva cortisol measurements feature multiple timepoints, data were summarized using four parameters that were then analyzed using the same ANCOVA model: area under the curve (AUCg), area under the curve incremental to the first timepoint considered as baseline (AUCi), maximum concentration (Cmax), and incremental maximum concentration (ICmax). For the autonomic parameters, the variables of interest were the changes from the pre-stressor value that was considered as baseline. These differences vs. baseline were analyzed using a linear mixed model with treatment, visit, treatment*visit, gender, DASS-42, and baseline (pre-stressor timepoint) as fixed and subject as random effects.

For endpoints not meeting the model assumptions of normality, a Box-Cox transformation was applied. In such cases, a back-transformation with bias adjustment was applied to obtain an estimate of the effect on the original scale. When models did not converge or model assumptions were severely violated, a Wilcoxon rank-sum test was used together with the Hodges–Lehmann estimator and an associated nonparametric 95% confidence interval.

In order to summarize the main findings of the whole study in a single descriptive figure, a principal component analysis (PCA) was performed on the difference between baseline and post-intervention of heart rate and PSS-14 as active variables and PSQI total score, PSQI subjective sleep quality score, HADS depression score, HADS anxiety score, cortisol acute response to experimental stress, cortisol awakening response, RMSSD at baseline (first period), RMSSD After 8 period, and DASS-42 baseline as passive variables. This PCA was performed on z-score-transformed variables.

The statistical significance level was fixed at 5%, without adjustments for multiplicity, due the exploratory nature of the study. All analyses were performed using SAS BASE 9.4/SAS STAT 15.1 on the SAS Life Science Analytics Framework (SAS LSAF, SAS Institute Inc., Cary, NC, USA) version 5.2.3.

## 3. Results

Demographic and clinical characteristics at enrollment were well-balanced across the 45 participants (probiotic *n* = 24; placebo *n* = 21) who completed the study, providing a Full Analysis Set (FAS, see Table 1).

### 3.1. Increased Abundance of BL NCC3001 in Stool Shows That the Probiotic Was Successfully Delivered

**Figure 2:**
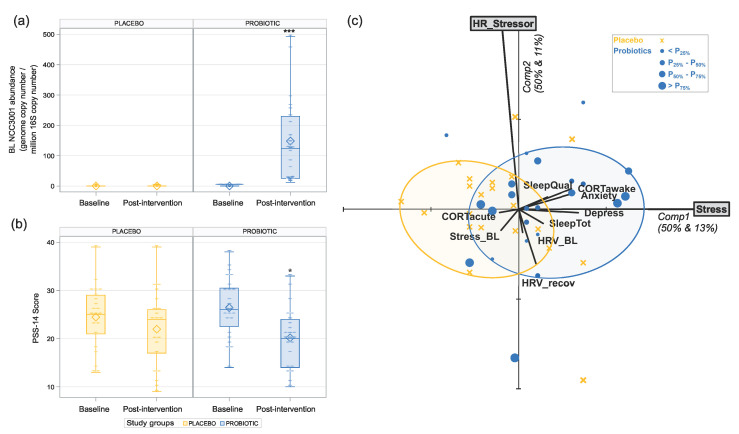
Effects of 6 weeks of intervention with *BL NCC3001* in healthy adults with self-reported mild-to-moderate stress. (**a**) Probiotic intervention significantly increased the abundance of *BL NCC3001* in stool. *** *p* < 0.001; (**b**) probiotic intervention significantly decreased the level of perceived stress as measured by PSS-14. Depiction of delta change post-intervention vs. baseline. * *p* < 0.05. (**c**) Principal Component Analysis (PCA) biplot on the z-scores of the baseline-to-post-intervention difference of heart rate (HR_Stressor) and PSS-14 (Stress) as active variables and all others as passive variables. This biplot simultaneously visualizes the correlation structure between active and passive variables as well as the multivariate positioning of individual subjects, with those in the probiotic intervention group being differentially marked according to the quartile they belong to in terms of detected probiotics. Smallest ellipses featuring 80% subjects are given for both groups.

Supplementation with *BL NCC3001* led to a substantial increase in the abundance of *BL NCC3001* in the probiotic group (model-based back-transformed mean difference: 118.90 *BL NCC3001*/10^6^ bacteria; *p* < 0.001) compared to the placebo group while at baseline; the quantification values of *BL NCC3001* were close to 0 for both groups and remained at 0 in the placebo group after treatment (Figure 2a).

### 3.2. Probiotic Intervention Reduced Perceived Stress Score

After 6-week intervention with *BL NCC3001*, the decrease in perceived stress in the probiotic group (21.4%) was significantly higher compared to that of the placebo group (−10.2%) (mean difference estimate of –3.278 between the probiotic and placebo groups with a 95% confidence interval (CI) of (–5.934, –0.622), *p* = 0.017; Figure 2b).

### 3.3. Impact of the Probiotic Intervention on Subjective Reports Independent of Acute Stress

At baseline, both the probiotic and placebo groups showed an average score under 8 on both the anxiety (HADS-A) and depression (HADS-D) subscales, with no significant difference between the groups (see Table 2).

Our population showed median, first, and third quartiles scores between 0 and 1 on the PSQI subscales (see Table 2 for a summary of the descriptive and inferential statistics). This suggests that most individuals did not have any signs of sleep disturbance. There was a significant improvement in subjective sleep in the probiotic group (5.25 higher odds risk ratio) compared to the placebo group (*p* = 0.037; Table 2). No improvements were observed in any of the other subscales of the PSQI (see Table 2 for a summary of the descriptive and inferential statistics).

**Figure 3:**
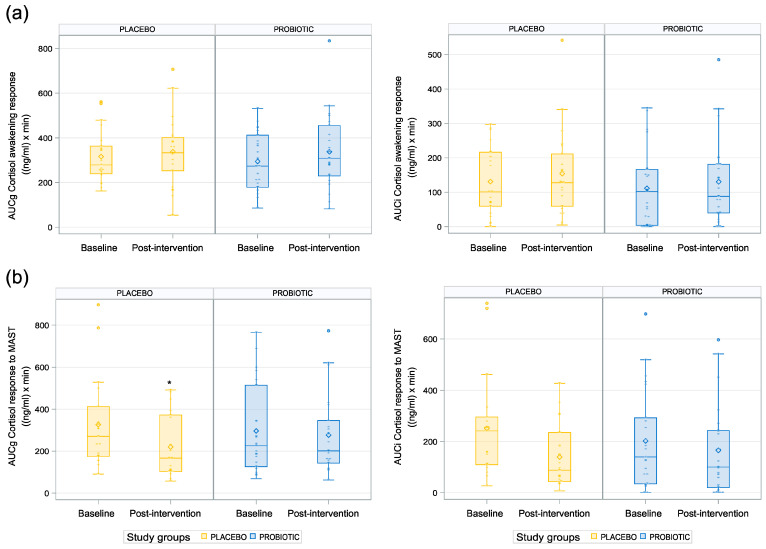
Differential cortisol response at waking and to acute stress: (**a**) cortisol awakening response; (**b**) cortisol response to acute stress. * *p* < 0.05.

To investigate the impact of the probiotic on the potential pathways of the gut–brain communication, the cortisol concentration upon waking was assessed as a marker of the HPA axis activity. Notably, we did not find any effect of the probiotic on the salivary cortisol awakening response compared to placebo (CAR AUCg: *p* = 0.812, CAR Cmax: *p* = 0.927; Table 3, Figure 3a).

We also investigated the gastrointestinal symptoms in this population experiencing mild-to-moderate stress using the Gastrointestinal Symptom Rating Scale (GSRS). No signs of gut discomfort were detected. The two experimental groups displayed comparable values (see Table 2).

### 3.4. Impact of the Probiotic Intervention on Stress and Autonomic Nervous System Parameters Linked to Acute Stress Response

The salivary cortisol concentration progressively increased up to 20 min following acute stress and then progressively decreased up to the last salivary sample at 40 min post-stressor. The cortisol stress response (AUCg and Cmax) was lower in the probiotic group compared to the placebo group at baseline, and the opposite pattern was observed post-intervention (Table 3). The inferential statistics revealed a higher AUC in the probiotic group compared to the placebo group post-intervention, but only in relation to ground, i.e., the measures related to absolute cortisol increases (*p* = 0.013; see Table 3, Figure 3b), while no difference was found in the AUCi. The estimate of this difference (assessed on the natural logarithm scale) reflected a 33.61 ng/mL×min difference in the AUCg between the probiotic group and the placebo group. Moreover, the results indicated a tendency toward a lower Cmax in the placebo group than in the probiotic group (*p* = 0.06, CI (−0.02, 0.71); see Table 3). Notably, this measurement is an absolute measure and does not correct for the participants’ baseline cortisol levels.

Relative to baseline, stress responses on autonomic parameters were characterized by an increase in HR, the LF peak, and SCL during the stressor (see Table 4) and an increased RMSSD immediately peaking after the stressor for both groups at both visits. From the inferential statistics, there were no clear pattern of group differences for the HRV data post-intervention (see Table 4). Similarly, no group difference was found for the electrodermal activity parameters.

**Figure 4:**
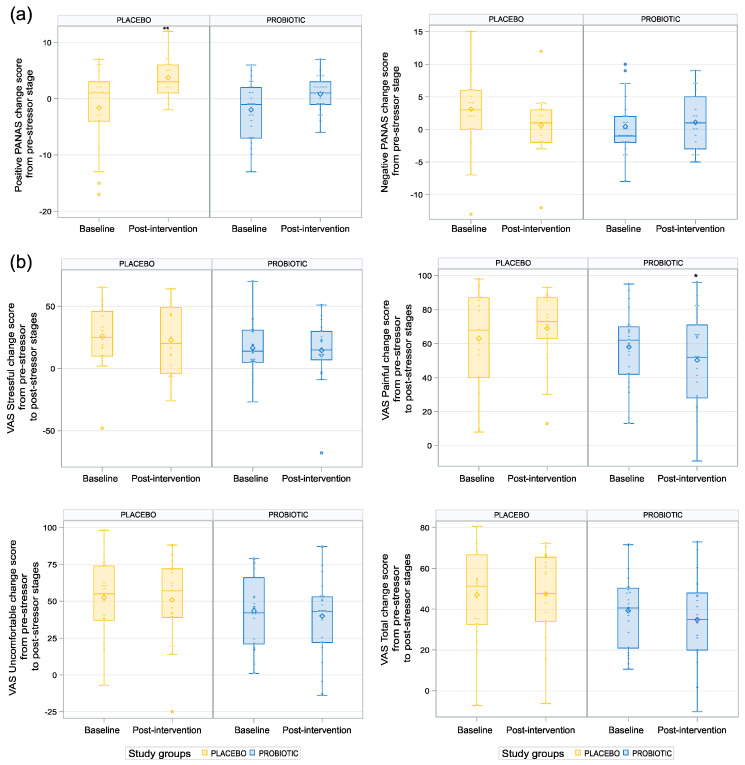
Impact of the probiotic intervention on subject mood assessments linked to acute stress response. (**a**) Placebo group significantly increased positive PANAS change score from pre-stressor stage with higher scores for the placebo group compared to the probiotic group. ** *p* < 0.01.(**b**) Lower values of VAS Painful change score from pre-stressor to post-stressor in the probiotic group. * *p* < 0.05.

We observed a statistically significant difference between the two groups post-intervention for the positive PANAS change score from the pre-stressor stage (*p* = 0.009; see Table 5 and Figure 4a), with higher scores for the placebo group than for the probiotic group, and for the VAS Painful change score from the pre-stressor stage, with lower values in the probiotic than placebo group (*p* = 0.02; see Table 5, Figure 4b). No difference was found between the groups for the negative PANAS change score, the STAI-6 change score, or any other VAS change score endpoints post-intervention.

### 3.5. Multivariate Analysis Revealed Positive Correlation between Stress Reduction and Reductions in Anxiety and Depression

Multivariate analysis revealed a positive correlation between the reduction in perceived stress with the reductions in anxiety (r = 0.32, *p* = 0.034) and depression (r = 0.34, *p* = 0.024) as well as the cortisol awakening response (r = 0.3, *p* = 0.047) (see Figure 2c). Notably, the ellipses featuring 80% of the subjects within each group are well-separated according to the first principal component, which visualizes the significant reduction in the stress perception in the probiotic group. Although the perceived stress and stress-related changes in the heart rate are almost independent, we noticed that the probiotic ellipse, which is located on the right side of the *y*-axis, was mainly populated with participants above the *x*-axis, showing that the probiotic group that decreased the most for perceived stress is also the one decreased the most for heart rate. Overall, multivariate analysis revealed a link between the reduction in perceived stress and the improvement in the psychological and physiological parameters.

## 4. Discussion

This exploratory trial evaluated the effect of *BL NCC3001* on the stress-related psychological and physiological parameters in healthy adults with mild-to-moderate psychological stress. The results support our hypothesis that supplementation with *BL NCC3001* can ameliorate perceived stress and, thus, provide a promising outcome for application in a population without mood disorders. This clinical observation was accompanied by better sleep quality in the probiotic group relative to the placebo group, and the substantial increase in the relative abundance of *BL NCC3001* in the stool samples from the probiotic group indicated that the probiotic was successfully delivered. Post hoc analyses further indicate that the improvement in perceived stress correlates with the reductions in anxiety, depression, and the cortisol awakening response.

Our main findings are in line with a recent meta-analysis assessing the effects of probiotics on stress in healthy subjects, showing that probiotics can reduce the subjective stress level in healthy volunteers without having a significant effect on the cortisol concentration [6]. A similar PSS-14 score difference between the placebo group and the probiotic group were shown in a parallel controlled study with 30 days of probiotic intervention with *Lactobacillus helveticus* R0052 and *Bifidobacterium longum* R0175 (3 × 10^10^ cfu/stick) on a subscale of 25 healthy adults with lower concentrations of 24 h urinary free cortisol at baseline [25]. Patterson et al. 2020 also reported effects on the PSS, particularly in female participants, with a decreased PSS score in the probiotic group (−1.00 point; −4.6%) and an increased PSS score in the placebo group (+2.36 points; +11.2%) following 5 weeks of intervention using 1.75 × 10^10^ cfu/day of *Lacticaseibacillus paracasei* Lpc-37 [26]. However, a study utilizing a *B. longum* strain for 1 week with a dose of 4 × 10^10^ cfu/day was unsuccessful in reducing self-reported perceived stress using this scale, albeit using a four-times-higher dose [27].

The baseline data obtained from subjective reports clearly showed a healthy population without suspicion of sleep disturbance. Although this initial status left limited space for improvement, we observed improved subjective sleep quality. This finding is in line with a recent study that showed an overall improvement in sleep quality and the duration of sleep using another *B. longum* strain, *B. longum* 1714, with a dose of 1 × 10^9^ cfu/day [28]. It has to be noted, however, that only male participants were assessed and that the age range was less broad (18–30 years).

The MAST procedure induced the anticipated increase in salivary cortisol [12,18], yet the results revealed an unexpected decreased response curve post-6-week intervention compared to baseline for both groups. Unlike other studies in healthy adults using a *B. longum* strain, we did not find a significant reduction in the cortisol stress response post-intervention by *BL NCC3001* relative to placebo [6,29,30]. A number of factors could account for this result. Different strains, such as *Lacticaseibacillus paracasei* Lpc-37 and *Lactobacillus plantarum* P8, and dosages of probiotic strains have different effects on the gut microbiome and the microbiota-gut–brain axis [26,30], and it is possible that the mechanism underlying stress reduction in the current study is not mediated by cortisol regulation. A recent study similarly reported a reduction in subjective stress in response to the MAST in the absence of changes in the cortisol response [31]. It is important to consider that cortisol release is a complex process that is influenced by many factors, in particular age [32] and gender [33], which were mixed in our population. Other factors not controlled for in this study, such as diet [34] and exercise habits [35], may also have influenced the cortisol release and contributed to the unexpected result. Unexpectedly, we observed that one of the measures of the absolute reduction in the salivary cortisol stress response following the MAST, the AUCg, was higher in the placebo group than in the probiotic group. Given the observed high interindividual variance in the cortisol response over time, it is also important to note that changes in the cortisol measures that take participants’ baseline into account, such as AUCi, which is more sensitive to temporal changes and interindividual trajectories when assessing repeated cortisol measures [36], were not significantly different between groups, so the results should, therefore, be treated with caution. In line with this, a previous study empirically showed that AUCi did not differ between low- and high-resilience people, but AUCg marginally differed due to differences at baseline [37]. At the subjective level, the MAST most notably induced an increase in negative mood scores on the PANAS and in ratings of stressfulness, painfulness, and discomfort in both groups for the two visits. The only self-reported measure suggesting a positive effect relative to the intervention was for painfulness.

The finding that participants in the placebo group increased their positive mood scores on the PANAS after the MAST stressor was unexpected but may be due to the participants in this group feeling relieved that the study procedures were over with, since these participants reported a higher pain experience.

In contrast to the *BL NCC3001* intervention in dogs [8], we did not observe effects on the autonomous nervous system, such as on HR or HRV, during the stress procedure. It is important to note that the study with dogs that displayed anxious behavior was based on a homogeneous population of dogs of the same breed, in a controlled environment with the same nutrient supply, which was in contrast to our clinical trial that displayed greater variability in a non-controlled environment. This heterogeneity among healthy volunteers may induce disparity in the results. Indeed, it was shown that factors such as age and gender can influence HRV, cortisol, and the skin conductance response [38]. Thus, further studies are needed to better understand the individual factors contributing to the observed effects.

One possible explanation for the lack of significant results on any other stressor-dependent subjective measures is that the sample size may not have been large enough to detect any differences.

To investigate whether the probiotic intervention led to an overall change across all assessed parameters, we conducted a multivariate analysis that revealed a positive correlation between stress reduction and reductions in anxiety and depression. This positive association is in line with findings that perceived stress may increase negative emotions such as anxiety and depression [39], which is not surprising, as these conditions are often interrelated and can exacerbate each other. While the mechanisms underlying these correlations are not fully understood, the microbiota-gut–brain axis is thought to play a critical role in the links between the gut microbiota, mood, stress, and brain health [2,40].

There are several mechanisms that could link the probiotic effect to the gut microbiome and host, leading to the reduction in perceived stress and the correlating benefits for anxiety and depression. For example, *L. plantarum* P8 (2 × 10^10^ cfu/day) was shown to increase the production of neurotransmitters or neuroactive compounds in the gut following 12 weeks of intervention [41], which was linked to improved mood and a reduction in stress and anxiety. Furthermore, the modulation of perceived stress may occur through alterations in gut hormones and gut peptides [42]. Another pathway includes neural connections such as the vagus nerve [43] that are associated with changes in HR and HRV, as observed by our previous research in dogs that displayed anxious behavior [8]; however, this was not observed in this clinical trial, possibly because of the higher heterogeneity in our population, as highlighted earlier. In addition, probiotics were also shown to modulate the production of short-chain fatty acids (SCFAs) by the gut microbiome, which have effects on neuro- and psychological functioning [44,45]. In this regard, we previously clinically showed that *BL NCC3001* modulates the metabolites of the microbiota–host co-metabolism [7]. Another possible mechanism to consider is the modulation of proinflammatory signaling, which can be found to be elevated in subjects facing psychological stress [46]. Dysbiosis of the gut microbiota was linked to increased inflammation, which was implicated in the development of mood disorders. Probiotics may also improve the function of the intestinal barrier, which can become compromised in individuals with stress symptoms [47,48]. A healthy intestinal barrier helps to prevent the entry of harmful substances such as toxins, pathogens, harmful bacterial products, and immune factors such as LPS into the systemic circulation [47]. These can trigger systemic inflammation and facilitate stress responses and mood disturbances in preclinical models, which were suggested to be an underlying mechanism in human disease [47,48]. Therefore, the mechanisms facilitating the effects of probiotics on stress and mood are likely multifaceted and involve a variety of different pathways and mechanisms. Moreover, effects can be strain specific. It is also important to consider other lifestyle factors that may influence stress and mood, such as diet, exercise, sleep, and social interaction [49]. Incorporating probiotics into a comprehensive approach for stress management that includes these other lifestyle factors may be the most effective way to reduce psychological stress and improve mood.

Overall, our results support the potential of specific probiotics being used to reduce perceived stress and will guide future clinical trial design to confirm the observed changes. Nevertheless, it is important to note that this exploratory clinical trial has several limitations. A larger, powered trial is warranted to confirm the findings of this exploratory trial, though the current findings can guide the design of any future confirmatory clinical studies in terms of effect size and expected variability. Moreover, the dosage and duration of probiotic supplementation play a key role in their effectiveness for reducing stress and improving mood. Some studies showed that higher doses of probiotics [26,30,50] or longer supplementation periods [28,51] may be needed to see significant improvements in mood, sleep, and stress reduction in healthy subjects. While we did see a mood improvement with *BL NCC3001* in subjects with IBS [7], the dosage or the duration of the intervention may not be large enough for a healthy population. It is also possible that this specific probiotic strain may require a population with higher mood alterations to evidence a clearer effect. Indeed, we did not observe any indication of a decline in mood in this healthy population. Furthermore, addressing anxiety in well-defined patient populations, e.g., IBS, [7] or in stressed animals [9] may have different neurological substrates compared to a healthy cohort. Moreover, it is conceivable that ecological factors in the gut microbiome played a role, the additional supplementation of a *B. longum* strain may not add a significant benefit to the existing metabolic networks of the resident *B. longum* strains within the gut microbiome, or the existing gut microbiome ecosystem did not allow any modulation of the metabolic pathways or their capacities. Exposure to chronic stress could disrupt the balance of the intestinal microbiota, so the daily intake of specific probiotics could then serve to prevent dysbiosis under certain conditions.

Our results show, for the first time, that long-term oral supplementation with *BL NCC3001* leads to a beneficial effect on stress relief and improves subjective sleep quality in a healthy adult population reporting moderate levels of psychological stress. In response to the Maastricht Acute Stress Test, momentary subjective assessments revealed a lower amount of pain experience in the probiotic group and a higher amount of relief at the end of the procedure in the placebo group, as reflected by the higher scores in the positive affect state. Multivariate analysis indicated that a reduction in perceived stress correlated with a reduction in anxiety, in depression, and in the cortisol awakening response after oral intervention. Larger, powered clinical trials are warranted to confirm the exciting findings on stress relief and sleep quality and provide further insights into the mechanisms underlying the stress-relieving and sleep-improving effect of *BL NCC3001*.

## Figures and Tables

**Table 1:** Demographic and clinical characteristics at enrollment (FAS). FAS, Full Analysis Set; BMI, Body Mass Index; DASS, Depression Anxiety and Stress Scale.

Parameter	Probiotic	Placebo	Total
N Age (years)	24 37.5 ± 10	21 40.7 ± 9.0	45 39.0 ± 9.6
BMI (kg/m^2^)	23.2 ± 2.5	22.8 ± 2.5	23.0 ± 2.5
Gender	13/11 (54/46%)	13/8 (62/38%)	26/19 (58/42%)
DASS-42 Score	19.1 ± 2.7	18.8 ± 2.9	19.0 ± 2.8
DASS-42 Stress Level (mild-to-moderate)	44/56%	46/54%	45/55%

**Table 2:** Psychological scores—independent of MAST. HADS-A, HADS Anxiety Score; HADS-D, HADS Depression Score. Inferential statistics: ^1^ nonparametric test (Wilcoxon rank-sum test), ^2^ model-based (log-scale). * *p* < 0.05. Data are expressed as mean ± SD or estimated with CI.

	Baseline		Post-Intervention, 6 w		6 w: Probiotic vs. Placebo Comparison		
Endpoint	Placebo	Probiotic	Placebo	Probiotic	Estimate	95% CI	*p* Value
PSS-14	24.5 ± 6.7	26.4 ± 6.1	22.0 ± 7.9	20.2 ± 6.0	−3.28	(−5.93, −0.62)	0.017 *
HADS-A	7.6 ± 3.9	7.0 ± 3.2	6.3 ± 3.5	5.2 ± 2.6	−0.65	(−2.05, 0.76)	0.358
HADS-D	4.6 ± 3.1	4.6 ± 3.2	4.2 ± 2.5	3.3 ± 2.7	−0.83	(−2.03, 0.38)	0.172
GSRS Reflux	1.3 ± 0.6	1.5 ± 0.9	1.3 ± 0.5	1.5 ± 0.9	0	(0, 0)	0.853 ^1^
GSRS Abdominal Pain	2.1 ± 1.1	1.9 ± 0.9	1.7 ± 0.7	1.6 ± 0.6	−0.04	(−0.24, 0.16)	0.676 ^2^
GSRS Indigestion	2.6 ± 1.2	2.4 ± 1.0	2.2 ± 0.9	2.0 ± 0.8	−0.04	(−0.20, 0.12)	0.577 ^2^
GSRS Diarrhea	1.6 ± 1.0	1.6 ± 0.9	1.6 ± 0.6	1.4 ± 0.4	−0.06	(−0.26, 0.13)	0.504 ^2^
GSRS Constipation	2.0 ± 1.2	1.8 ± 0.9	1.7 ± 0.9	1.4 ± 0.5	−0.12	(−0.32, 0.08)	0.238 ^2^
PSQI Subjective Sleep Quality	1.2 ± 0.4	1.1 ± 0.6	1.1 ± 0.3	0.8 ± 0.5	0	0	0.037 ^1^*
PSQI Sleep Latency	1.0 ± 0.7	1.0 ± 0.6	0.8 ± 0.6	0.8 ± 0.6	0	0	0.923 ^1^
PSQI Sleep Duration	0.2 ± 0.4	0.2 ± 0.5	0.3 ± 0.5	0.2 ± 0.4	0	0	0.391 ^1^
PSQI Habitual Sleep Efficiency	0.4 ± 0.7	0.3 ± 0.6	0.3 ± 0.6	0.2 ± 0.4	0	0	0.358 ^1^
PSQI Sleep Disturbances	1.2 ± 0.4	1.0 ± 0.4	1.0 ± 0.4	1.0 ± 0.0	0	0	0.554 ^1^
PSQI Use of Sleeping Medication	0.0 ± 0.2	0.1 ± 0.3	0.0 ± 0.0	0.1 ± 0.3	0	0	0.182 ^1^
PSQI Daytime Dysfunction	1.1 ± 0.6	1.1 ± 0.5	0.7 ± 0.6	0.7 ± 0.6	0	0	0.862 ^1^
PSQI Total	5.2 ± 1.8	4.8 ± 2.2	4.3 ± 1.7	3.8 ± 1.4	−0.38	(−1.13, 0.37)	0.308 ^1^

**Table 3:** Effect of *BL NCC3001* on cortisol stress response and cortisol awakening response. AUCg, area under the curve following acute stress or as cortisol awakening response (ng/mL) × min); AUCi, area under the curve incremental to the first timepoint considered as baseline (ng/mL) × min); CAR, cortisol awakening response (ng/mL); Cmax, maximum concentration; ICmax, incremental maximum concentration. Inferential statistics: ^1^ model-based (log-scale); ^2^ model-based (sqrt-scale). * *p* < 0.05. Data are expressed as mean ± SD or estimated with CI.

	Baseline		Post-Intervention, 6 w		6 w: Probiotic vs. Placebo Comparison		
Endpoint	Placebo	Probiotic	Placebo	Probiotic	Estimate	95% CI	*p* Value
Acute Stress AUCg	326.4 ± 217.6	296.3 ± 211.2	219.7 ± 151.1	276.7 ± 185.0	0.39	(0.09, 0.69)	0.013 ^1^*
Acute Stress AUCi	251.2 ± 199.7	201.9 ± 189.6	139.5 ± 125.5	165.1 ± 174.7	1.58	(−1.29, 4.45)	0.271 ^1^
Acute Stress Cmax	10.6 ± 7.6	9.4 ± 7.2	6.9 ± 5.3	8.2 ± 6.3	0.35	(−0.02, 0.71)	0.06 ^1^
Acute Stress ICmax	9.3 ± 7.3	7.8 ± 6.9	5.5 ± 4.9	6.1 ± 6.3	0.21	(−0.32, 0.75)	0.422 ^1^
CAR AUCg	315.9 ± 117.1	294.6 ± 126.3	337.7 ± 156.1	337.8 ± 166.7	0.31	(−2.34, 2.97)	0.812 ^2^
CAR AUCi	130.9 ± 100.1	111.4 ± 110.1	154.1 ± 126.2	130.4 ± 121.4	−0.84	(−4.18, 2.49)	0.611 ^2^
CAR Cmax	7.4 ± 2.7	7.1 ± 3.1	7.9 ± 4.1	7.8 ± 3.9	−0.11	(−2.60, 2.37)	0.927
CAR ICmax	4.1 ± 2.6	3.5 ± 3.1	4.8 ± 3.9	4.0 ± 3.3	0.07	(−0.48, 0.62)	0.795 ^1^

**Table 4:** Physiological responses to MAST. Effect of *BL NCC3001* on the autonomic parameters of heart rate, heart rate variability, and skin conductance. Inferential statistics: ^1^ period1-centered, ^2^ model-based (log-scale), ^3^ nonparametric test (Wilcoxon rank-sum test), ^4^ model-based (sqrt-scale, shift = 13), and ^5^ model-based (sqrt-scale, shift = 13). Data are expressed as mean ± SD or estimated with CI.

		Baseline		Post-Intervention, 6 w		6 w: Probiotic vs. Placebo Comparison		
Endpoint	Period	Placebo	Probiotic	Placebo	Probiotic	Estimate	95% CI	*p* Value
Mean HR (1/min)	Before	73.9 ± 9.5	73.9 ± 8.6	73.6 ± 11.6	74.7 ± 8.2	0.94	(−3.20, 5.08)	0.649
	During 1	77.5 ± 10.1	76.6 ± 9.5	78.1 ± 11.7	77.2 ± 8.0	−1.85	(−5.25, 1.55)	0.284 ^1^
	After 8	69.8 ± 7.5	66.4 ± 8.4	67.6 ± 9.2	68.8 ± 6.6	0.53	(−2.89, 3.95)	0.760 ^1^
RMSSD (ms)	Before	42.6 ± 32.6	46.3 ± 33.8	33.2 ± 11.6	64.0 ± 101.3	0.17	(−0.22, 0.56)	0.382 ^2^
	During 1	59.2 ± 50.5	77.4 ± 82.0	37.7 ± 22.6	52.9 ± 39.2	2.63	(−2.31, 7.72)	0.225 ^1,3^
	After 8	48.1 ± 18.3	89.6 ± 93.7	63.9 ± 70.3	60.4 ± 35.1	3.38	(−13.96, 14.32)	0.610 ^1,3^
Sympathetic-Vagal Balance = LF/HF	Before	2.9 ± 2.0	3.3 ± 3.4	3.6 ± 2.4	2.8 ± 2.4	−0.26	(−0.74, 0.23)	0.29 ^2^
	During 1	3.2 ± 1.9	3.4 ± 1.99	3.2 ± 2.0	3.8 ± 2.9	0.22	(−0.03, 0.47)	0.08 ^1,4^
	After 8	4.6 ± 4.3	3.5 ± 3.96	3.2 ± 2.3	2.9 ± 2.7	0.07	(−0.18, 0.32)	0.592 ^1,4^
LF (Hz)	Before	0.1 ± 0.03	0.1 ± 0.02	0.1 ± 0.02	0.1 ± 0.02	−0.003	(−0.02, 0.01)	0.644
	During 1	0.1 ± 0.03	0.1 ± 0.02	0.1 ± 0.03	0.1 ± 0.02	−0.005	(−0.02, 0.01)	0.603 ^1^
	After 8	0.1 ± 0.02	0.1 ± 0.02	0.1 ± 0.03	0.1 ± 0.02	−0.007	(−0.03, 0.01)	0.471 ^1^
HF (Hz)	Before	0.2 ± 0.07	0.3 ± 0.06	0.2 ± 0.06	0.2 ± 0.07	0.02	(−0.03, 0.05)	0.453
	During 1	0.2 ± 0.07	0.2 ± 0.08	0.2 ± 0.06	0.2 ± 0.08	0.01	(−0.03, 0.06)	0.529 ^1^
	After 8	0.2 ± 0.05	0.2 ± 0.05	0.2 ± 0.06	0.2 ± 0.06	0.01	(−0.03, 0.05)	0.661 ^1^
Skin Conductance (microS)	Before	1.5 ± 2.1	1.5 ± 1.8	1.7 ± 2.3	1.9 ± 3.2	−0.11	(−0.90, 0.67)	0.77 ^2^
	During 1	3.4 ± 3.5	3.5 ± 2.6	3.5 ± 3.9	3.8 ± 4.9	−0.03	(−0.21, 0.16)	0.786 ^1,5^
	After 8	3.5 ± 4.5	2.1 ± 2.4	2.6 ± 3.0	3.0 ± 4.2	−0.01	(−0.2, 0.18)	0.911 ^1,5^

**Table 5:** Psychological responses to MAST. Effect of *BL NCC3001* on positive and negative moods, on anxiety score following MAST, and on Visual Analogue Scale (VAS) from pre- to post-stressor. All scores are displayed as change score from pre-stressor stage. * *p* < 0.05, ** *p* < 0.01. Data are expressed as mean ± SD or estimated with CI.

	Baseline		Post-Intervention, 6 w		6 w: Probiotic vs. Placebo Comparison		
Endpoint	Placebo	Probiotic	Placebo	Probiotic	Estimate	95% CI	*p* Value
Positive PANAS	−1.6 ± 7.1	−2 ± 5.2	3.7 ± 3.7	0.8 ± 3.1	−2.87	(−5.01, −0.73)	0.009 **
Negative PANAS	3.1 ± 6.4	0.4 ± 4.2	0.6 ± 4.5	1.1 ± 4.3	1.18	(−1.51, 3.86)	0.38
STAI-6 State	−0.6 ± 1.3	−0.9 ± 1.5	0 ± 1.4	−0.5 ± 1.4	−0.43	(−1.27, 0.40)	0.301
STAI-6 Trait	−0.2 ± 1.2	−0.4 ± 0.8	−0.6 ± 1.3	−1 ± 1.4	−0.43	(−1.23, 0.37)	0.286
VAS Stressful	26.1 ± 26.1	16.4 ± 21	22.7 ± 26.5	14.6 ± 24.6	−3.42	(−17.98, 11.15)	0.638
VAS Painful	63 ± 29.9	58.1 ± 21.8	69.1 ± 21.8	50.2 ± 26.8	−16.5	(−30.26, −2.74)	0.02 *
VAS Uncomfortable	52.4 ± 26.2	43.4 ± 23.5	50.9 ± 29.1	39.9 ± 28.0	−10.04	(−28.69, 8.62)	0.282
VAS Total	47.1 ± 22.4	39.3 ± 17.5	47.6 ± 20.7	34.9 ± 21.1	−8.83	(−19.21, 1.56)	0.093

## Supplementary Materials

The following supporting information can be downloaded at https://www.mdpi.com/article/10.3390/nu15143122/s1. Table S1: Schedule of the assessments at baseline and post-intervention.
