# Supplementary material for: Bifidobacterium longum subsp. longum Reduces Perceived Psychological Stress in Healthy Adults: An Exploratory Clinical Trial
Source: Nutrients. 2023 Jul 13;15(14):3122. doi: 10.3390/nu15143122 (PMC10383821; doi:10.3390/nu15143122)
Supplement: Supplementary file 1 [file nutrients-15-03122-s001.zip › nutrients-2445306-supplementary.pdf]

Supplementary Materials

Supplementary Table S1. Schedule of the assessments at baseline and post-intervention.

		Duration (Total ≈ 100′) *	Description of Baseline and Post-Intervention	Timepoints
Continuous autonomic measurements	Pre-Stress	30′	Providing stool and waking saliva samples<br>Placement of the portable device for autonomic measurements (calibration)<br>Compliance questionnaire<br>Questionnaires (PSS, HADs, GRSR, and PSQI)<br>Instructions on the procedures	
		5′	Rest and filler activities<br>Saliva sample t-20	Before
		5′	PANAS scale<br>STAI-6	
	Stressor	5′	MAST instructions and anticipation phase<br>VAS subjective perception before stressor	During 1
		10′	Maastricht Acute Stress Test	
			HIT (90 s)	
			MA (45 s)	During 2
			HIT (60 s)	During 3
			MA (60 s)	During 2
			HIT (60 s)	During 3
			MA (90 s)	During 2
			HIT (90 s)	During 3
			MA (45 s)	During 2
			HIT (60 s)	During 3
			Saliva sample t + 0 **	During 2
			VAS subjective perception during stressor	
	Post-Stress/Recovery	5′	VAS subjective perception after stressor<br>PANAS scale<br>STAI-6	After 1
		5′	Saliva sample t + 5<br>Rest	After 2
		5′	Saliva sample t + 10<br>Rest	After 3
		5′	Rest	After 4
		5′	Saliva sample t + 20<br>Rest	After 5
		5′	Rest	After 6

5′	Saliva sample t + 30<br>Rest	After 7
5′	Rest	After 8
2′	Saliva sample T + 40	
5′	Blood sampling	

* Small variations in completion time may be due to the time taken to read the task summary instructions and/or to complete self-reported assessments, to read the instructions on the computer screens, and/or to collect saliva samples; ** t0 refers to end of stressor; GSRS, Gastrointestinal Symptom Rating Scale; HADS, Hospital Anxiety and Depression Scale; PANAS, Positive and Negative Affect Schedule; PSS, Perceived Stress Scale; PSQI, Pittsburgh Sleep Quality Index questionnaire; VAS, Visual Analog Scale; STAI-6, State Trait Anxiety Inventory 6-item; HIT, Hand Immersion Trial; MA, mental arithmetic.
